# Cell cycle and cell death are not necessary for appressorium formation and plant infection in the fungal plant pathogen *Colletotrichum gloeosporioides*

**DOI:** 10.1186/1741-7007-6-9

**Published:** 2008-02-14

**Authors:** Iris Nesher, Sima Barhoom, Amir Sharon

**Affiliations:** 1 Department of Plant Sciences, Tel Aviv University, Tel Aviv 69978, Israel

## Abstract

**Background:**

In order to initiate plant infection, fungal spores must germinate and penetrate into the host plant. Many fungal species differentiate specialized infection structures called appressoria on the host surface, which are essential for successful pathogenic development. In the model plant pathogen *Magnaporthe grisea *completion of mitosis and autophagy cell death of the spore are necessary for appressoria-mediated plant infection; blocking of mitosis prevents appressoria formation, and prevention of autophagy cell death results in non-functional appressoria.

**Results:**

We found that in the closely related plant pathogen *Colletotrichum gloeosporioides*, blocking of the cell cycle did not prevent spore germination and appressoria formation. The cell cycle always lagged behind the morphogenetic changes that follow spore germination, including germ tube and appressorium formation, differentiation of the penetrating hypha, and *in planta *formation of primary hyphae. Nuclear division was arrested following appressorium formation and was resumed in mature appressoria after plant penetration. Unlike in *M. grisea*, blocking of mitosis had only a marginal effect on appressoria formation; development in hydroxyurea-treated spores continued only for a limited number of cell divisions, but normal numbers of fully developed mature appressoria were formed under conditions that support appressoria formation. Similar results were also observed in other *Colletotrichum *species. Spores, germ tubes, and appressoria retained intact nuclei and remained viable for several days post plant infection.

**Conclusion:**

We showed that in *C. gloeosporioides *the differentiation of infection structures including appressoria precedes mitosis and can occur without nuclear division. This phenomenon was also found to be common in other *Colletotrichum *species. Spore cell death did not occur during plant infection and the fungus primary infection structures remained viable throughout the infection cycle. Our results show that the control of basic cellular processes such as those coupling cell cycle and morphogenesis during fungal infection can be substantially different between fungal species with similar lifestyles and pathogenic strategies.

## Background

Fungal spores are resting structures that efficiently disseminate the fungal organisms from which they originate. Therefore, spores are programmed to germinate only under appropriate conditions, which vary between species according to lifestyle and nutritional requirements. In plant pathogenic fungi, germination is frequently stimulated by plant-specific signals, such as plant-derived compounds or the physicochemical properties of the plant surface. In the genus *Colletotrichum*, spores are affected by self-inhibitory compounds that prevent germination in dense populations [1,2], but germination and appressorium formation can be triggered by specific external signals such as cuticular waxes or hard hydrophobic surfaces [3-5]. Spore germination in soil-borne fungi is enhanced by root exudates, whereas in rust fungi the direction of germ tube growth and appressoria formation are sensitive to the properties of the leaf surface [6,7].

Fungal spores must complete several developmental stages on the host surface before they can penetrate into host tissues. The sequence of events includes activation of metabolism, germ tube initiation, a short period of polar growth coupled with a limited number of cell divisions, polar growth arrest, and differentiation of an appressorium. Since the surface of a leaf lacks most nutrients, completion of the pre-penetration development depends primarily on the spore's endogenous resources and involves degradation and recycling of stored lipids, carbohydrates, and nitrogen sources [8]. It is presently unclear whether the limited growth of the pathogen on the host surface is restricted by developmental programs, by nutritional limitations, or by both.

Studies of the model plant pathogen *Magnaporthe grisea *showed that spore carbon and lipids sources are degraded during germination or translocated into the developing appressorium [9,10]. Upon contact with a host surface, spores of *M. grisea *form a short germ tube and then differentiate a dome-shaped appressorium that generates high turgor pressure by accumulating high levels of glycerol [11,12]. Early studies showed that the spore and germ tube collapsed following appressorium formation and it has been suggested that the spore and germ tube cytoplasm move into the appressorium [12,13]. Thines et al [9] showed a mass transfer of spore carbohydrates and lipid bodies into the young appressorium that is controlled by PMK1 MAP kinase and cAMP pathways. Recent work has presented evidence that spores of *M. grisea *undergo autophagic cell death following mitosis and appressorium formation [14]. Completion of mitosis and the autophagy cell death were found essential for appressorium formation and appressorium mediated penetration, respectively; mutants that were unable to undergo mitosis could not produce appressoria, while autophagy mutants *mgatg8 *and *mgatg1 *differentiated appressoria, but were unable to penetrate into the host plant [14,15]. Similarly, in *Colletotrichum lindemuthianum*, mutants in CLK1 (a homolog of MgATG1) are also unable to cause disease owing to the inability of appressoria to penetrate the host cuticle [16]

*Colletotrichum gloeosporioides *f. sp. *aeschynomene *is a hemibiotroph plant pathogen infecting the legume plant *Aeschynomene virginica*. It shares similar lifestyles and infection strategies with *M. grisea*, particularly during the early stages of pathogenesis. Spores quickly germinate on the host surface, form a short germ tube, and then differentiate well-developed, melanized appressoria. These appressoria generate high turgor pressure and then differentiate infection pegs that penetrate the plant cuticle by mechanical force. We previously showed that *C. gloeosporioides *can germinate in two distinct ways: on plants or on a hydrophobic surface spores undergo mitosis and then develop a single germ tube [3]. This process is initiated immediately following induction of germination and terminates within less than 4 h, with the formation of appressoria. In rich media, spores germinate as described in *Aspergillus *[17]; upon induction, the spore swells, and after several hours it forms a germ tube and then a second germ tube develops from the other side of the spore. Such a germination process does not lead to the formation of an appressorium and these spores have reduced pathogenicity. We have defined these two germination processes as 'pathogenic' and 'saprophytic', respectively [3]. The two germination processes in *C. gloeosporioides *are regulated by different signaling cascades: saprophytic germination is enhanced by cAMP, whereas pathogenic germination is cAMP-independent. Similar to *M. grisea*, cAMP is required later for appressoria formation.

In this work we have characterized the sequence of events occurring during pathogenic spore germination using a nucleus green fluorescent protein (GFP)-tagged strain. We found that inhibition of the cell cycle did not prevent spore germination, and that mitosis was not required for appressorium differentiation. We also found that spores, hyphae, and appressoria retained intact nuclei and were viable for over 72 h post plant inoculations. These results are in sharp contrast to the situation described in *M. grisea *[14]. Importantly, our results indicate that even in closely related plant pathogens that use similar infection strategies to invade plant tissue, regulation of the infection process is very different.

## Results

### Nuclear division and germ tube formation

**Figure 1:**
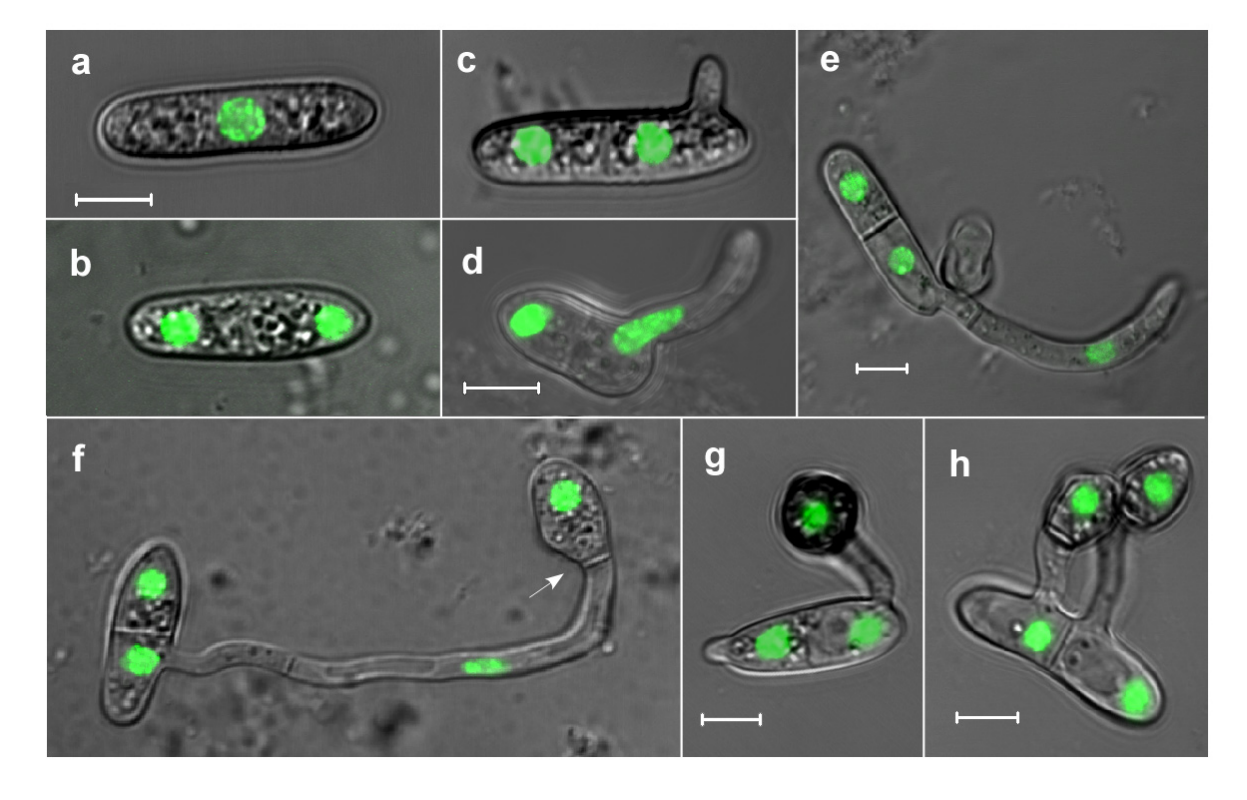
**Nuclear division and morphogenesis during early stages of pathogenic germination**. Spores of strain H1-13 were germinated on a slide in pea extract: (a) resting spore; (b) first nuclear division (75–90 min); (c) germ tube formation (90–120 min); (d,e) germ tube elongation and second nuclear division (150–180 min); (f) appressorium formation and third nuclear division (4–6 h, arrow points to the septum between the germ tube and appressorium); (g, h) formation of the second germ tube and appressorium (7–9 h). Pictures represent sequence events and are projections of optical sections. The scale bar is 5 μm.

**Figure 2:**
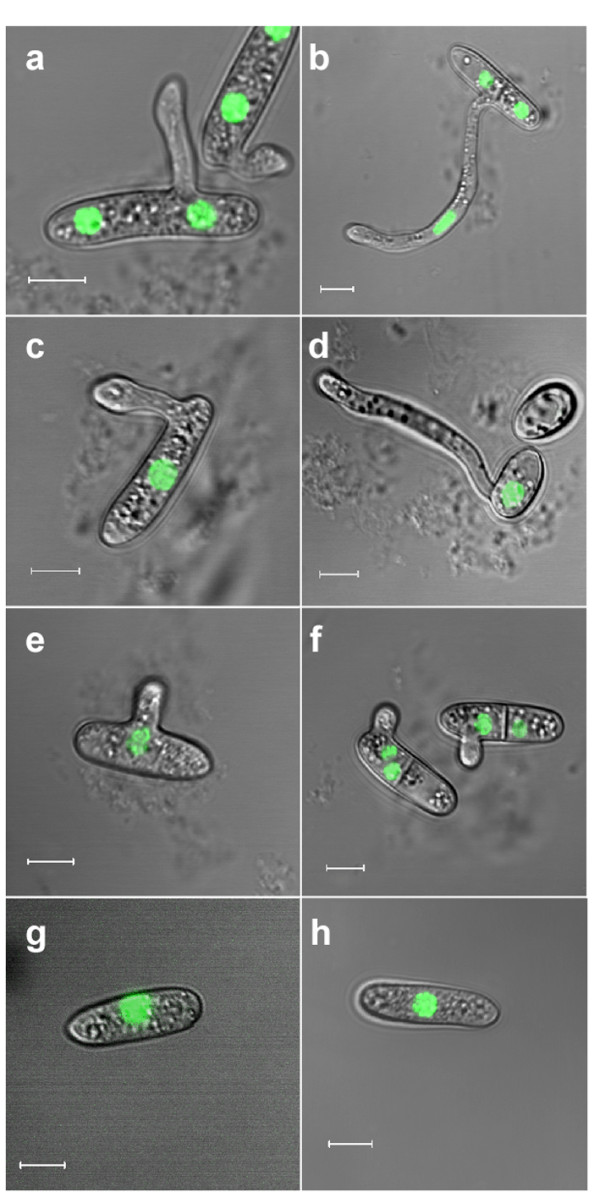
**Effect of inhibitors on cell cycle and development**. Spores of strain H1-13 were germinated on a slide in PE (left 3 h; right 6 h): (a,, b) no treatment; (c, d) HU; (e, f) benomyl; (g, h) LatA. The scale bar is 5 μm.

We have generated transformants of *C. gloeosporioides *expressing Histone1-eGFP fusion protein (H1-GFP) that label nuclei with a strong eGFP fluorescence. These transformants had wild-type morphology, and showed normal development and pathogenicity. We used isolate H1-13 to characterize nuclear division following induction of pathogenic germination (Figure 1). Results were confirmed periodically by comparison with 4'-6-diamidino-2-phenylindole (DAPI) staining of the wild-type strain. The number of nuclear divisions was recorded and correlated with cell divisions and germ tube formation. The cell cycle resumed instantly following induction, and the first mitosis took place within approximately 90 min. The time between the first and second cell cycles was similar, with the second mitosis occurring after 180 min. Treatment of spores with hydroxy urea (HU), which prevents the G_1_/S transition, completely inhibited nuclear division, resulting in cells with a single nucleus (Figure 2c,d). These results indicate that the spores are specifically arrested at G_1_, but would germinate without delay under appropriate conditions.

The first nuclear division always occurred before germ tube formation. A single germ tube was first visible after 2 h. At this time point, spores have already completed their first mitosis leading to two uni-nuclear cells separated by a septum (Figure 1c and Additional file 1). The spore nucleus located near the site of emergence of the germ tube continued to divide, whereas cell cycle and morphogenesis of the adjacent spore cell were arrested. On a hydrophobic surface or on plants, elongation of the germ tube was arrested after two to four nuclear divisions followed by the formation of an appressorium at the end of the germ tube (Figure 1f). A second germ tube occasionally formed around 7 h after inducing germination. This second germ tube originated from the quiescent spore cell which resumed growth (Figure 1g,h).

### Spore germination can occur without mitosis

The linkage between cell cycle and morphogenesis was investigated using cell cycle blockers. HU-treated spores germinated readily, and although germination was somewhat delayed, the final germination rates were similar to the germination rates without the inhibitor (Table 1). As expected, nuclei of HU-treated spores did not divide, thus indicating that the cell cycle was arrested (Figure 2c). Despite the lack of nuclear division, the germ tube continued to elongate for several hours and reached the same size as germ tubes that developed by untreated spores (Figure 2b,d). Thus, HU completely prevented nuclear division in the spores but had almost no effect on germination and germ tube growth. Spores treated with benomyl (causes disassembly of the microtubule cytoskeleton, thereby blocking the G_2_/M transition and nuclei separation [18]) also had high germination rates (Table 1). However, benomyl-treated spores formed only a short germ tube before growth was arrested (Figure 2e,f). To test the possible effect of polar growth on the cell cycle, we treated spores with latrunculin A (LatA), which disrupts actin polymerization, thereby preventing polarized growth. As expected, LatA treatment completely blocked germination, but at the same time it also prevented nuclear division (Table 1 and Figure 2g,h).

**Table 1:** Effect of HU, benomyl, and LatA on spore germination. Spores were germinated on glass slides in PE. The percentage of spore germination was determined after 3 and 6 h. HU data is the average of four independent experiments. Benomyl and LatA experiments were repeated three times with two repeats in each experiment. Similar results were obtained in each of the three independent experiments.

**Treatment**	**Germination (%)**	
	
	**3 h**	**6 h**
H_2_O (control)	89 ± 7.5	93 ± 6
HU	46 ± 25	85 ± 6
EtOH (control)	89	97
Benomyl	65 ± 2	87 ± 1
DMSO (control)	92	98 ± 1
LatA	0	0

### Nuclear division and appressorium formation

**Figure 3:**
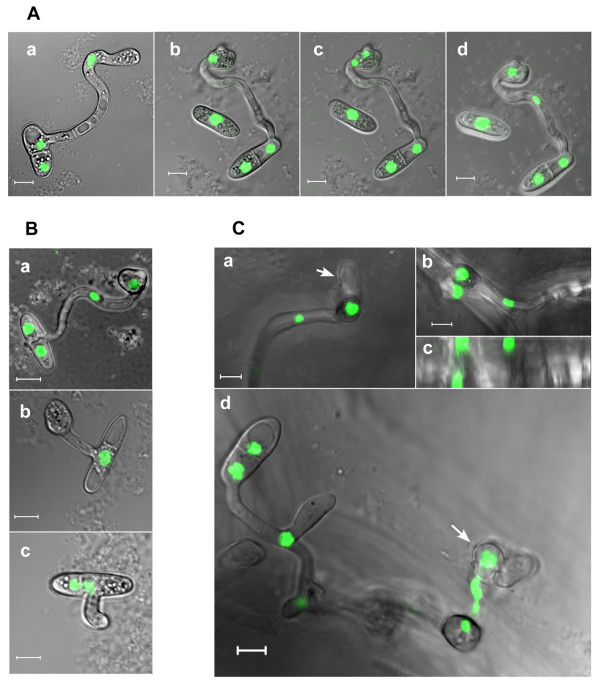
**Nuclear division and morphogenesis during appressorium formation and plant penetration**. (A) Appressorium morphogenesis. (a) Growth arrest and swelling of the hyphal tip (beginning of appressorium differentiation). Note that the nucleus is still in the hypha out of the incipient appressorium. (b-d) Time lapse of nuclear division within the appressorium: (b) the nucleus migrates from the hypha to the appressorium neck; (c) the nucleus divides within the appressorium neck; (d) one nucleus remains inside the appressorium and the other moves back into the hypha. (B) Effect of inhibitors on appressorium formation: (a) no treatment; (b) HU (fully developed appressorium develops without nuclear division); (c) benomyl. (C) Spores of strain H1-13 were inoculated onto onion epidermis. Pictures were taken after 24 h. (a) A hypha and an appressorium develop on the surface; a primary hypha is formed inside the plant under the appressorium. Note that the primary hypha is formed before nuclear division (arrow). (b, c) Nuclear division occurs in the appressorium after primary hypha formation. A single nucleus remains inside the appressorium: (b) projections of optical sections; (c) side view of the same sample. (d) A picture showing the spore from which infection originated, a hypha that developed on the leaf, an appressorium, and the underlying developing primary hyphae with several nuclei (arrow). Note that the spore and appressorium, which are on top of the onion epidermis, contain intact nuclei. Picture is a projection of optical sections. The scale bar is 5 μm.

On plants or on hydrophobic surfaces, the spores of *C. gloeosporioides *germinate in pathogenic mode and develop appressoria [3]. Although the external conditions that induce appressoria formation are known, it is unclear what type of endogenous signals might determine when the switch from polarized growth to tip swelling will occur. We followed appressoria formation and correlated it with nuclear divisions to assess a possible association between the number of cell cycles and appressorium formation. On a hydrophobic surface, the single germ tube underwent two to four nuclear divisions before the tip started to swell and develop an appressorium (Figure 1f). Following appressorium formation and the beginning of maturation (characterized by the accumulation of melanin), the nucleus migrated from the germ tube into the junction between the germ tube and the newly formed appressorium. Subsequently, this nucleus divided, one of the resulting nuclei migrated back into the germ tube, and a septum was formed, separating the appressorium from the adjacent germ tube (Figure 3A). HU treatment inhibited nuclear division in the elongating hyphae, but had no effect on appressoria formation; similar numbers of fully developed appressoria were formed in HU-treated and control spores (Table 2 and Figure 3Bb). We repeated these experiments with four additional *Colletotrichum *species and all of them were able to produce appressoria without mitosis. Although germination rates were variable between the different species, the percentage of germ tubes that formed appressoria were only slightly reduced by HU treatment and were similar to the rate of appressoria formation in *C. gloeosporioides *(Table 2). Despite the formation of normal appressoria, the HU-treated spores failed to penetrate onion epidermis (data not shown). Benomyl-treated spores developed only a short germ tube and growth was stopped before appressoria formation (Figure 3Bc), possibly as a result of the role of microtubules in long-range cargo delivery. Thus, nuclear division is dispensable for growth and development throughout germination and appressoria formation; however, further development and plant penetration were prevented.

**Table 2:** The effect of HU on germination and appressoria formation in five different *Colletotrichum *species. Fungi were germinated on the surface of a Petri dish in water (*C. magna, C. coccodes, C. acutatum*) or in PDB medium (*C. gloeosporioides, C. musae*). Spore concentration was 10^5^/ml in all species. Percentage germination and appressoria formation were determined after 7 h (*C. gloeosporioides, C. coccodes*), 9 h (*C. acutatum, C. musae*), or 13 h (*C*. *magna*). In each sample 150 spores were counted, with two independent samples per experiment. Appresoria percentage is the percentage of germ tubes that formed appressoria.

**Species**	**Germination (%)**		**Appressoria (%)**	
	
	**H**_2_**O**	**HU**	**H**_2_**O**	**HU**
*C. gloeosporioides*	100	100	83.5 ± 7.5	77 ± 3
*C. magna*	91.5 ± 6	45.5 ± 12.5	95 ± 3	75 ± 10
*C. acutatum*	80 ± 15	73 ± 3	47 ± 1	33 ± 2
*C. musae*	94 ± 2	86.5 ± 6.5	35.5 ± 5.5	25.5 ± 7.5
*C. coccodes*	63.5 ± 15	26.5 ± 22	66 ± 1	50 ± 25

### Nuclear division occurs in appressoria after the completion of plant penetration

We monitored the development of appressoria and infection hyphae on onion epidermis. Following the migration of a nucleus into the appressorium, a septum formed that separated the appressorium from the germ tube (Figure 1f). During appressorium maturation, which can last up to 24 h, no further nuclear division could be detected in the appressoria, suggesting that cell cycle progression was arrested. Upon plant penetration, nuclear division within the appressorium was resumed, one of the nuclei migrated into the penetration hypha, whereas the other nucleus remained inside the appressorium (Figure 3Cb,c). Further mitoses followed as primary hyphae developed inside the plant tissue (Figure 3Cd). This result is in sharp contrast to *M. grisea*, in which the nucleus migrates from the appressorium into the penetrating hypha and then undergoes mitosis just before the development of the invasive hypha [14].

### Spores and appressoria remain viable following plant invasion

**Figure 4:**
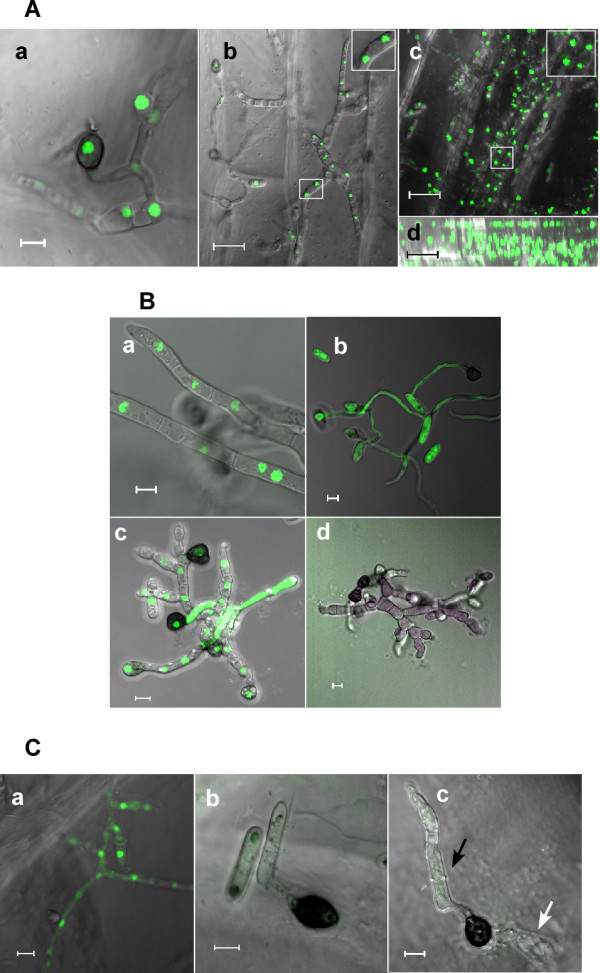
**Viability of spores and appressoria after plant infection**. (A) Spores of strain H1-13 were inoculated onto onion epidermis. (a) After 24 h post inoculation all mycelial cells that developed on the surface of the onion epidermis contain intact nuclei. (b) After 48 h post inoculation the mycelium and appressoria on the leaf surface still retain intact nuclei. Pictures represent the scan of the surface without optical sections. (c, d) Projection of optical sections showing the hyphae on and inside the leaf 72 h post inoculation: (c) top view; (d) side view of the same image. Upper nuclei line is on the leaf surface. (B) Spores were germinated on a slide with PE. (a) Untreated hyphae of strain H1-13 showing intact nuclei. (b) Spores and hyphae of wild-type strain stained with FDA (positive staining indicates viable cells). (c) Spores of strain H1-13 were germinated and then treated with lovastatin, which induces apoptosis. Picture was taken 24 h after treatment. Note the abnormal development of the hyphae and smearing of the GFP signal, which indicates nuclei disintegration. (d) Spores of the wild-type strain were germinated and then treated with lovastatin. The sample was stained with FDA 24 h after lovastatin application. (C) TUNEL assay of mycelium on the onion epidermis. Spores of the wild-type strain were inoculated onto the onion epidermis. TUNEL staining was performed 48 h post infection. (a) DNAse-treated sample (positive control). (b) Control of a sample that was incubated only with labeling solution without the terminal transferase (negative control). (c) Picture showing a spore (black arrow), a mature appressorium and underlying primary hyphae (white arrow) stained with TUNEL. Lack of staining indicates lack of PCD (viable cells). The scale bar for (b-d) in (A) is 20 μm; for all others it is 5 μm.

We noticed that GFP-labeled nuclei in the spores, hyphae, and appressoria remained intact following infection of onion epidermis and pea leaves (Figure 4A). The retention of an intact H1-GFP signal has been considered as evidence of cell viability [14]. To verify the correlation of the H1-GFP nuclear signal with cell viability, we treated mycelium with lovastatin, which induces apoptotic-like cell death in *C. gloeosporioides *[19], and checked cell viability and the H1-GFP signal after 24 h. Control cells of the H1-GFP strain developed normal mycelia with intense nuclear GFP signal (Figure 4Ba). Wild-type cells, which do not express GFP stained positive with fluorescein diacetate (FDA), confirming that these cells were indeed viable (Figure 4Bb). In the lovastatin-treated hyphae, development stopped within hours and cells were swollen and misshapen (Figure 4Bc). These hyphae contained cells in different stages: cells with an intact nucleus, cells in which the nuclear signal was lost and the GFP signal was uniformly distributed within the entire cell, and cells that completely lost the GFP signal (Figure 4Bc). Lovastatin-treated cells of a wild-type strain did not stain with FDA, indicating that these hyphae were non-viable (Figure 4Bd). We concluded from these experiments that the nuclear GFP signal in the H1-GFP cells is indicative of viable cells, but that the initiation of cell death processes might occur before loss of the GFP signal, as indicated by the loss of FDA staining. Cells in which the GFP signal spread in the cytoplasm or completely disappeared are no longer viable. To further rule out possible apoptotic cell death of the early infection structures, we inoculated onion epidermis with a wild-type strain and conducted a terminal uridine deoxynucleotidyl transferase dUTP nick end labeling (TUNEL) assay after 48 h, at which time the fungus penetrated the onion cells and developed primary as well as secondary hyphae. No staining was observed in the spore, hyphae, or appressoria, indicating that the fungus growing on top of the epidermis remained viable throughout the infection process (Figure 4C). These results indicate that the spores do not die following differentiation of appressoria, and that all of the primary infection structures formed on the leaf remain viable even when secondary hyphae develop, which represent the final, necrotrophic stage of the disease.

## Discussion

Plant pathogenic fungi devised various strategies and mechanisms to infect plants. However, regardless of the pathogenic lifestyle, the initial stages of infection are common to most fungi: spores germinate on the host surface, undergo a short period of polarized growth, and then develop appressoria, which direct the growth into the plant tissues. Since the plant surface is poor in nutrients, it is assumed that fungi must utilize the spore's endogenous reserves to quickly move from the plant surface into the inner tissues. Various signals such as cutin monomers or surface physical features are known to induce appressoria formation, but it is unclear at which point post germination this switch from polar growth to swelling of the tip will occur. The short period of growth on the host surface is characterized by tight coupling of morphogenesis and cell cycle progression. On surfaces that support appressoria formation such as on plants or on a hydrophobic surface, appressoria will form on short germ tubes after a few nuclear divisions. On surfaces that do not support appressoria formation the germ tube will elongate and undergo many more nuclear and cell divisions. Thus, the number of cell divisions is not used by the fungus to determine the stage of appressorium formation without an appropriate surface signal. In this work we have studied the association between nuclei division and morphogenesis during pathogenic spore germination, appressorium formation, and appressorium-mediated plant penetration using a *C. gloeosporioides *transformant expressing Histone1-eGFP fusion protein.

Analysis of nuclear divisions revealed that mitosis was initiated immediately following exposure to germination-inducing signals. The first mitosis was observed 90 min after induction, which is similar to the elapsed time between the first and second mitoses. This suggests that in *C. gloeosporioides*, growth and cell division are inhibited in spores, but the spores are not dormant and can readily resume growth without a preceding adaptation stage upon receiving the proper signals. The growth inhibition is attributed to self-inhibitory compounds in the spore cell wall since removal of these substances by washing of the spores can enhance spore germination even without specific induction [1,2]. The instant mitosis triggered during pathogenic germination differs from the pattern observed during saprophytic germination in *C. gloeosporioides *and other fungal species in which breaking of dormancy and isotropic swelling precedes mitosis and growth [20]. In *Candida albicans*, cells that grow to high densities are arrested in G_1_. When diluted to lower densities the cells resume growth, but the first cell cycle is characterized by an extended G_1 _phase, which is about 70 min longer than the G_1 _phase in dividing cells [21]. In *Aspergillus nidulans*, spores are dormant and germination is preceded by an early stage that lasts several hours and includes activation of metabolism and isotropic swelling [17,22]. In *C. gloeosporioides*, saprophytic germination includes an initial period during which spores start swelling, but are neither growing nor dividing [3]. In contrast, pathogenic germination has no adaptation stage; mitosis is tightly linked with germination response and seems to occur simultaneously or prior to cell growth.

We used a pharmacological approach in attempting to separate between the cell cycle and cell growth. Treatment of cells with HU always prevented the first nuclear division. This result indicates that in *C. gloeosporioides *the spores were arrested at G_1_, similar to the situation in other species [18,23,24]. The continuation of germination without mitosis is also in agreement with results from other species. In *F. graminearum *nuclear division is not required for spore germinating in rich medium [25]. Under similar conditions, *A. nidulans *spore germination is tightly coordinated with nuclear division, but these two processes can be uncoupled under less favorable conditions [22]. In *Aspergillus fumigatus*, cell cycle and morphogenesis are not interdependent, and it has been suggested that similar to yeast, these two processes run in parallel but share common checkpoints [26]. Taken together, it seems that in most cases initiation of germination is independent of the cell cycle.

Blocking of mitosis in germinating spores of *M. grisea *using HU or by genetic intervention also lead to germination without nuclear division; however, appressoria formation was prevented [14]. Similar results were reported in *C. trancatum*, where inhibition of the second round of DNA synthesis did not affect spore germination but blocked appressoria formation [27]. In contrast, *C. gloeosporioides *spores continued to grow for at least 7–8 h in presence of HU, forming normal numbers of well-developed appressoria. Treatment of spores with benomyl also had no effect on germination rates, but growth was retarded at an earlier stage before appressoria formation. The early growth retardation following benomyl treatment is attributed to the role of microtubules in growth rather than to the lack of nuclei separation [28]. These results suggest that in *C. gloeosporioides *all stages of pathogenic germination, including appressoria formation, are independent of mitosis, unlike *M. grisea *and *C. trancatum *in which appressoria formation and pathogenic development depend on completion of mitosis [14,27]. Several other *Colletotrichum *species that we tested also produced high rates of appressoria in presence of HU, which were only slightly lower than the percentage of appressoria formation in untreated spores (Table 2). We noted that all of these species have small, unicellular spores whereas both *M. grisea *and *C. trancatum *have larger, three cells spores. At this point we do not know whether this structural difference has anything to do with the differences that we found in the regulation of cell cycle and appressoria formation.

We previously suggested a model in which plant signals bypass the effect of the self-inhibitory compounds and activate both mitosis and morphogenesis [3]. Our current results suggest that induction of germination is unrelated to the induction of the cell cycle. This implies that either these two processes run in parallel and are simultaneously induced by the same signals, or that they might respond to different stimuli. In order to address these different possibilities, we used LatA to block cell growth and determined the effect on nuclear division. Unlike HU, which only blocked mitosis, LatA completely blocked both spore germination and nuclear division. When LatA was removed by washing of the spores, germination was resumed, showing that the effect was not a result of the toxicity of LatA but rather of the specific disruption of the actin cytoskeleton (data not shown). Thus, prevention of polar growth also leads to cell cycle arrest.

That morphogenetic development is necessary for continuation of the cell cycle is further supported by our observations that morphological changes always occurred before nuclear division. Following the first cell division, a germ tube emerged from one of the spore cells, and only then did the nucleus in this cell divide and migrate into the growing germ tube (Additional file 2). Similarly, the nucleus in the germ tube adjacent to the appressorium divided only when the appressorium was fully developed and it divided again only after the mature appressorium developed a penetrating hypha. Thus, in *C. gloeosporioides *morphogenesis always occurs before mitosis and the cell cycle depends on completion of the preceding morphogenetic stage.

According to these new results, we propose that plant signals specifically activate germination and that the cell cycle is induced only after initiation of germination. According to this model, the self-inhibitory compounds only inhibit growth, whereas cell cycle arrest is not directly affected by these compounds. This sequence of events is opposite from that in *M. grisea*, in which mitosis must be completed before appressorium formation, and the nucleus within the appressorium does not divide but migrates from the appressorium through the penetration peg into the primary hyphae [14]. These differences are surprising, especially because *M. grisea *and *C. gloeosporioides *share a very similar infection strategy. Recently, Kankanala et al. [29] reported that *M. grisea *infects plant cells by moving biotrophic hyphae from one cell to another. This mode of infection differs from the infection mode of the true hemibiotroph *Colletotrichum *species, including *C. gloeosporioides *which use biotrophic hyphae to invade a few cells and then differentiate necrotrophic hyphae that kill the host cells [30]. These differences, together with the difference in regulation of the early stages of infection, indicate that despite the apparent similarities in the sequence of events leading to pathogenesis, these fungi must have evolved very different regulatory mechanisms to control pathogenesis.

Another striking difference between *C. gloeosporioides *and *M. grisea *was our finding that the spores and appressoria of *C. gloeosporioides *remained viable throughout the infection cycle, unlike the spores of *M. grisea*, which were subjected to autophagy cell death soon after appressorium formation [14]. This result is especially surprising since the unicellular spores of *C. gloeosporioides *are much smaller and therefore contain fewer resources than the three-celled spores of *M. grisea*. In several other fungal species it has been shown that spore components, and especially lipids and carbohydrates are recycled during germination [8]. These works suggest that recycling of the spore components during germination is necessary for progression of the early stages of fungal development. In *M. grisea *mutants in the MAP kinase PMK1 and mitosis-defective mutants that are unable to form appressoria keep growing on the leaf surface without loosing viability [14]. Additional *M. grisea *mutants also show this phenomenon, including G proteins and cAMP pathway mutants [31-33]. Moreover, in other fungi, mutants in similar genes were also able to develop considerable amounts of mycelia on the leaf surface [34,35]. In *C. gloeosporioides*, spores that are germinated in rich medium do not develop appressoria and are not infective, but keep developing on the leaf surface [3]. Collectively, these results show that when regulation of early pathogenesis is interrupted, fungal spores are capable of growing on the leaf surface for much longer periods of time than the very limited number of nuclear divisions, which normally precede appressoria formation. Therefore, the number of nuclear divisions prior to appressoria formation is not restricted by nutrient availability but rather, the limited growth of germ tubes before appressoria formation seems to constitute a common element in the regulation of early fungal pathogenesis. When the signal for growth arrest is missing, owing to mutations or lack of appropriate signals (e.g. on a hydrophilic surface or in rich medium), polar growth continues and mycelium develops. The endogenous nutrients within spores should be sufficient to support production of this biomass, or fungi must be able to absorb nutrients without plant penetration. Although we presently have no answers to these questions, we assume that both possibilities might occur. The final behavior of the spore, either for life or death, seems to be of variable importance; in some cases such as in *M. grisea*, it might be important, whereas in other cases such as in *C. gloeosporioides*, programmed cell death does not occur and is not required for plant infection.

## Conclusion

Spores of *C. gloeosporioides *are arrested at G_1_, but are not dormant. Germination signals activate cell polarization, and although growth and cell cycle occur in parallel, appressoria formation is independent of mitosis. Other *Colletotrichum *species with unicellular spores can also form appressoria without mitosis, suggesting that this phenomenon is not unique to *C. gloeosporioides*. Another striking difference between *C. gloeosporioides *and *M. grisea *is our finding that the spores and appressoria of *C. gloeosporioides *remain viable throughout the infection cycle. Thus, despite the apparent similarities in pathogenic development, these fungi must have evolved very different regulatory mechanisms to control appressoria formation and host infection. The limited growth of germ tubes constitutes a common element in the regulation of early fungal pathogenesis. The development on the leaf surface is restricted by regulatory signals, but is not limited by nutrients availability, suggesting either that the endogenous nutrients are sufficient to support production of a relatively large fungal biomass, or that fungi can obtain external nutrients even before plant penetration.

## Methods

### Fungi, plants, and growth conditions

*Colletotrichum gloeosporioides *f. sp. *aeschynomene *3.1.3 wild-type and transgenic strains were used. Emerson's YpSs (EMS) solid media and pea extract (PE) were prepared as described previously [36]. Inoculation experiments were conducted with onion epidermis. All of the experiments were conducted in a growth chamber at 28°C.

### Fungal transformation

Germinating spores were transformed as described previously [36]. Transgenic strains that express the Histone1-eGFP fusion protein were obtained by co-transformation of the pMF-280 plasmid [37], which carries the *Histone1-eGFP *gene fusion under the *CCG-1 *promoter, and the p57-GPD-HPH plasmid, which carries the *HPH *resistance gene under the *A. nidulans GPDA *promoter.

### Germination

Spores were harvested from 5-day-old EMS plates in ddH_2_O, then counted, and diluted to 10^6 ^spores/ml in PE. Germination assays were performed on hydrophobic (Petri dish) or hydrophilic (glass slide) surfaces, using 30-, 50-, or 100-μl droplets. Plant inoculation assays were performed with 10-μl droplets of 10^5 ^spores/ml water suspensions with 0.01% Tween-20 (Sigma).

### Cell cycle and cytoskeleton

HU (Sigma) was prepared as a 1 M stock solution in ddH_2_O and used for blocking of cell cycle, at a final concentration of 30 mM. Benomyl (Sigma) was prepared as a 1 mg/ml stock solution in 95% ethanol and used to inhibit nuclei separation and blocking of the cell cycle, at a final concentration of 5 μg/ml. LatA (Calbiochem) was prepared as a 1 mM stock in dimethylsulfoxide (DMSO) and used to disrupt actin microfilament polymerization, at a final concentration of 5 μM. Compounds were diluted in PE medium.

### Apoptosis

Apoptosis was induced by treating the fungus with lovastatin (Calbiochem). Lovastatin was prepared as a 6-mM stock solution in ethanol and added to spores two hours after induction of germination, at a final concentration of 250 μM. Apoptosis was determined by the TUNEL assay using the *In Situ *Cell Death Detection kit, Fluorescein (Roche Diagnostics, Germany) as described previously [19].

### Staining and microscopy

FDA staining was performed as described previously [3]. Fluorescent and light pictures were taken using LSM 510 confocal microscope. Excitation and emission wavelengths for FDA and GFP were 493/510 nm and 488/505 nm, respectively.

## Authors' contributions

IN designed the study, carried out the cell cycle and morphogenesis analyses, and helped drafting the manuscript. SB performed the viability assays and helped drafting the manuscript. AS conceived the study, participated in its design and coordination, and drafted the manuscript. All authors have read and approved the final version of the manuscript.

## Supplementary Material

Additional file 1 **Time lapse microscopy of first nuclear division during spore germination (S1.wmv)**. Note that the first nuclear division occurs before germ tube formation. Pictures were taken every 3 min, at 40× magnification. Click here for file

Additional file 2 **Time lapse microscopy of second nuclear division during conidial germination (S2.wmv)**. Second nuclear division occurs during germ tube elongation. Pictures were taken every 3 min, at 25× magnification. Click here for file
